# Survey of Primary Care Physicians’ Screening and Treatment Practices for Prediabetes in Saudi Arabia

**DOI:** 10.7759/cureus.21475

**Published:** 2022-01-21

**Authors:** Faisal Aljehani, Abdullah Alsulaiman, Abdulrahim Alqarni, Fahad Almutairi, May Samkari

**Affiliations:** 1 College of Medicine, Department of Internal Medicine, University of Jeddah, Jeddah, SAU; 2 Health Program and Chronic Disease Adminstration, Public Health Administration at Saudi Ministry of Health, Jeddah, SAU

**Keywords:** primary care, diabetes, risk factors, survey research, knowledge

## Abstract

Background

Prediabetes is defined as a condition in which glucose levels do not fulfill the criteria for type 2 diabetes mellitus (T2DM), indicating that the patient is at an increased risk of developing T2DM. The risk of developing T2DM can be decreased by adequately managing prediabetes. This study aimed to assess screening and therapeutic approaches to prediabetes among primary care physicians in Saudi Arabia because there is little contemporary data available on this topic.

Methodology

A cross-sectional study was performed among primary care physicians in Saudi Arabia. The participants completed a validated online survey questionnaire via Google Forms. Data collected included participants’ demographic information, knowledge of T2DM risk factors, and opinions and beliefs on prediabetes management.

Results

In total, 155 primary care physicians responded to the questionnaire; 51% were male, 18.7% worked in Riyadh City, and 81.3% specialized in family medicine. Most study respondents (71.9%) were residents, and 64.5% worked for the Ministry of Health. Overall, 93.5% of the respondents had completed part of their postgraduate training in Saudi Arabia. Moreover, 27.7% of the respondents were aware of all nine risk factors associated with T2DM. The correct fasting glucose and hemoglobin A1c ranges for the diagnosis of prediabetes were identified by 50% and 43.6% of participants, respectively. Most respondents believed lifestyle modification and metformin to be the most effective management approaches to prediabetes, whereas lack of motivation toward lifestyle changes was deemed to be a major barrier.

Conclusions

We found significant gaps in primary care physicians’ knowledge regarding prediabetes in Saudi Arabia, contributing to underscreening of the condition and undertreatment. Identifying these gaps is essential for focussing educational endeavors toward primary care physicians.

## Introduction

According to the American Diabetes Association’s (ADA) guidelines, prediabetes refers to glucose levels that do not fulfill the criteria for type 2 diabetes mellitus (T2DM). Individuals with prediabetes have an increased risk of T2DM onset, cardiovascular pathologies, and all-cause mortality [1,2]. In addition, prediabetes increases the risk of developing T2DM and correlates with an annual progression of 5-10% toward T2DM [3].

Prediabetes is associated with impaired fasting glucose and/or impaired glucose tolerance. Recent data indicate that more than one-third of adults in affluent countries have prediabetes [4]. Fasting glucose concentrations of 100-125 mg/dL, hemoglobin A1c (HbA1c) titers of 5.7-6.4%, and two-hour post-stimulus glucose levels of 140-199 mg/dL are used to identify prediabetes. Behavioral lifestyle changes and medications are useful in preventing the progression of prediabetes to T2DM [1].

Future health problems can be prevented with early identification and treatment of prediabetes and T2DM. However, many individuals with metabolic disorders remain undiagnosed, which can be attributed in part to the knowledge and clinical practices of primary care physicians (PCPs). Through the detection and management of prediabetes, PCPs play a crucial role in diabetes prevention. Understanding the level of PCPs’ knowledge concerning prediabetes is key for successful screening initiatives and the commencement of suitable management interventions [5,6]. However, little is known about PCPs’ knowledge either in Saudi Arabia or globally [7].

Most patients with prediabetes do not receive evidence-based preventive care from their PCPs [8,9]. Systemic barriers hinder physicians’ ability to provide the best evidence-based practice, such as performance measures, insurance reimbursement, cultural expectations, and a lack of tools and staffing resources [10].

In a study conducted in the Al-Hassa region of Saudi Arabia in 2010, PCPs had substantial gaps in their knowledge regarding screening, diagnosing, and managing prediabetes [11]. Another study performed in 2020 in the Al-Qassim region reported that PCPs’ knowledge, attitudes, and practices were suboptimal [12]. To date, no national study on this topic has been published in Saudi Arabia; however, surveys from the United States have exposed the limited awareness among PCPs regarding the risk factors that should be screened to detect prediabetes, the diagnostic laboratory parameters for this condition, and management guidelines once prediabetes is recognized [6,7].

This study aims to close the knowledge gap regarding PCPs’ screening and treatment practices for prediabetes in Saudi Arabia.

## Materials and methods

Study design and settings

This was a cross-sectional study. PCPs living and practicing in Saudi Arabia were eligible for inclusion in the study. PCPs were invited using email and social media, for example, Twitter or WhatsApp, to complete a modified validated online survey questionnaire via Google Forms. The original questionnaire was adapted from a similar study conducted in the United States [5].

Study participants

The study targeted PCPs in Saudi Arabia who were invited to access the study questionnaire link and respond to its items. The link was shared over their social media platforms, including email, WhatsApp, Twitter, etc. No incentive was offered to the participants.

Data collection and study instruments

A modified validated questionnaire was employed [5], and the responses were collected via Google Forms. The questionnaire was open for responses between May 1, 2021, to June 25, 2021. The questionnaire is provided in the Appendix section. The questionnaire included items regarding participants’ demographics, specialty, training, organization, knowledge about diabetes risk factors, diabetic and prediabetic fasting glucose (mg/dL), HbA1c (%) diagnostic cut-off, minimum weight loss, physical activity for prediabetics, and guidelines for diabetes screening. Items on knowledge, practice, and beliefs regarding prediabetes management were assessed. The questionnaire also included items about participants’ opinions regarding the importance of identifying prediabetes in their patients, at risk of developing diabetes, use of lifestyle modifications and metformin to reduce diabetes risk, barriers to lifestyle modification for patients with prediabetes, interventions to improve management and treatment of prediabetes, barriers to the adoption of the ADA guidelines, and suggestions regarding using metformin in patients with prediabetes. Additionally, we assessed participants’ responses to prescribing metformin for patients with prediabetes and if the ADA guidelines for patients with prediabetes helped manage patients with prediabetes.

Data analysis

The data were entered, organized, tabulated, and analyzed using SPSS Statistics for Windows, version 26.0 (IBM Corp., Armonk, NY, USA). Demographic and socioeconomic data were tabulated and expressed as the frequency and percentage of the total participants. We used tables to report participant responses to survey questions as the frequency and percentage of the total participants. Figures illustrate the frequency distribution of the different variables.

Ethical consideration

Ethical approval for the study was obtained from the Research Ethics Committee of the University of Jeddah. The questionnaire was approved and validated by experts before it was sent to the PCPs. Data from the research were used only for research purposes. Consent was obtained from participants who agreed to provide their responses for use in our research. We did not collect or store any identifiable data.

## Results

In total, 155 PCPs responded to the online survey. The participants’ demographics, specialty, training, and work organization are listed in Table 1. Overall, 51% of the participants were males, 98.7% were Saudi, 18.7% were from Riyadh city, and 81.3% specialized in family medicine. Most of the participants (71.9%) were residents; 64.5% worked for the Ministry of Health and 93.5% had completed part of their postgraduate training in Saudi Arabia.

**Table 1:** Distribution of surveyed participants according to their demographics, specialty, training, and work organization (n = 155).

Variable	Number (%)
Gender	
Male	76 (49)
Female	79 (51)
Nationality	
Non-Saudi	2 (1.3)
Saudi	153 (98.7)
City	
Al Jouf	3 (1.9)
Al Bahah	1 (0.6)
Al Madinah	16 (10.3)
Al-Ahsa	3 (1.9)
Aseer	12 (7.7)
Dammam	14 (9)
Hafar Albatin	1 (0.6)
Hail	3 (1.9)
Jazan	14 (9)
Jeddah	25 (16.1)
Makkah	14 (9)
Najran	1 (0.6)
Northern	2 (1.3)
Qassim	4 (2.6)
Qatif	1 (0.6)
Riyadh	29 (18.7)
Tabuk	7 (4.5)
Taif	5 (3.2)
Specialty	
Family medicine	126 (81.3)
General practitioner (without advanced training)	15 (9.7)
Internal medicine	11 (7.1)
Obstetrics/gynecology	3 (1.9)
Current level of training	
Consultant	7 (4.5)
General practitioner (without advanced training)	14 (9)
Resident	113 (71.9)
Specialist	21 (13.5)
Type of organization you work with now	
Military	9 (5.8)
Ministry of Health	100 (64.5)
Ministry of Health, Military	3 (1.9)
Ministry of Health, Private	2 (1.3)
National Guard	9 (5.8)
National Guard, Military	1 (0.6)
Private	4 (2.6)
University	20 (12.9)
University, Military	1 (0.6)
University, Ministry of Health	5 (3.2)
University, Private	1 (0.6)
Country/countries where you did any level of your postgraduate training	
Canada	2 (1.3)
Saudi Arabia	145 (93.5)
Saudi Arabia, Canada	1 (0.6)
Saudi Arabia, United States	3 (1.9)
Saudi Arabia, United States, Canada, United Kingdom	1 (0.6)
Saudi Arabia, United States, United Kingdom, Germany	1 (0.6)
United States	1 (0.6)
United States, Canada	1 (0.6)

**Figure 1:**
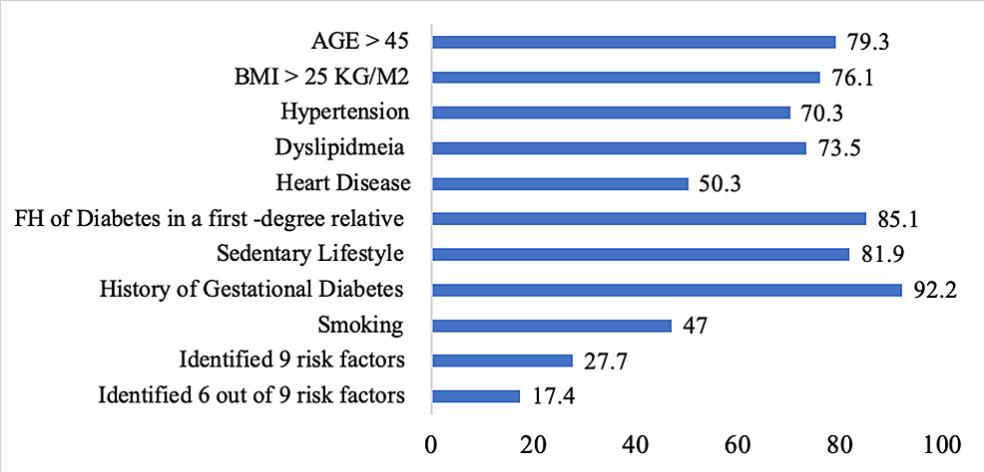
Distribution of risk factors for diabetes identified by study participants. BMI: body mass index; FH: family history

Participants were asked to identify diabetes risk factors. A history of gestational diabetes (92.2%), a family history of diabetes in a first-degree relative (85.1%), and a sedentary lifestyle (81.9%) were the most commonly known risk factors (Figure 1). Only 27.7% of the study participants were aware of all nine risk factors for T2DM mentioned in the survey.

**Figure 2:**
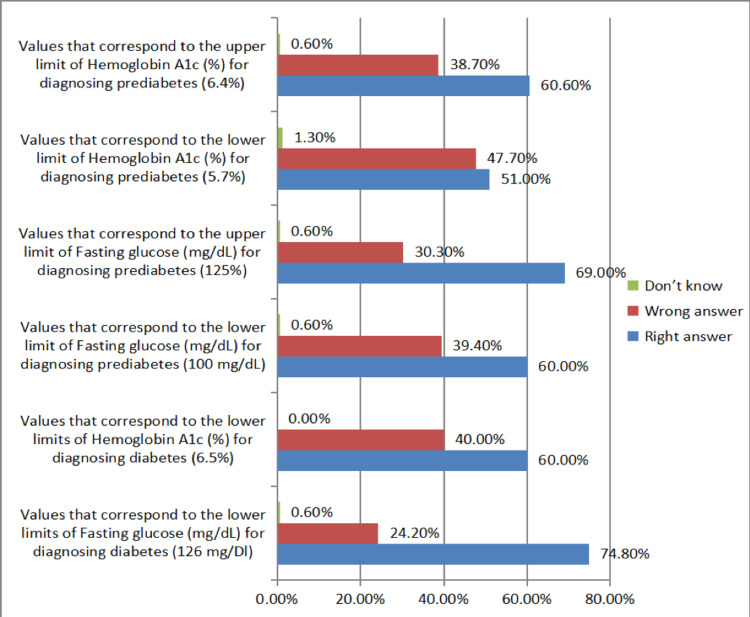
Distribution of participants based on identifying correct upper and lower limits of the laboratory criteria for the diagnosis of diabetes and prediabetes.

**Figure 3:**
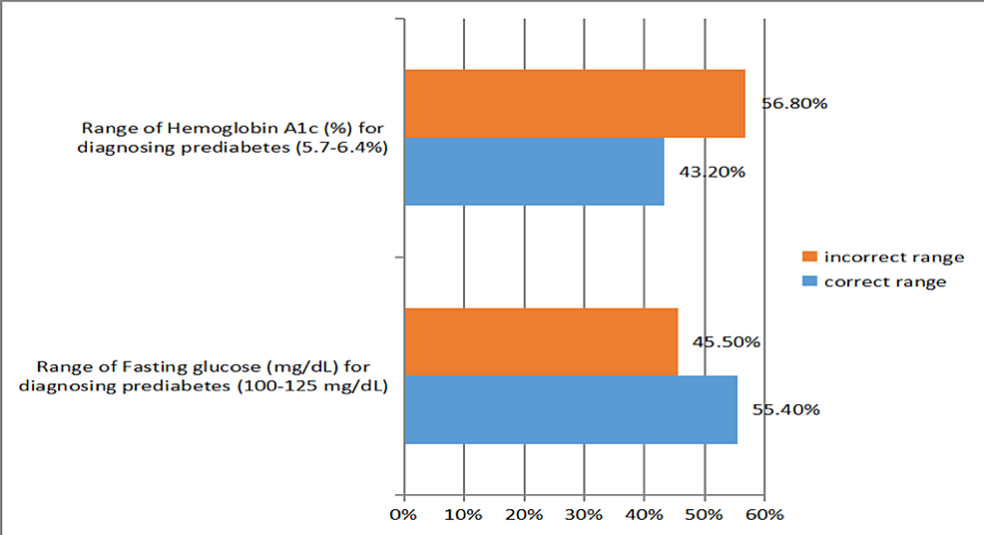
Percentage distribution of participants who were able to identify the correct ranges for fasting glucose and hemoglobin A1c for the diagnosis of prediabetes.

Regarding the diagnosis of diabetes, 74.8% and 60% of the participants were aware of the lower limits of fasting glucose (126 mg/dL) and HbA1c (6.5%), respectively. Moreover, 60% and 68% were able to identify the lower (100 mg/dL) and upper (125 mg/dL) limits, respectively, of fasting glucose for the diagnosis of prediabetes, respectively. Additionally, 51% and 60.6% could correctly identify the lower (5.7%) and upper (6.4%) limits of HbA1c, respectively, for the diagnosis of prediabetes (Figure 2). Overall, 55.5% of the participants identified the correct range of fasting glucose (100-125 mg/dL), and 43.6% were aware of the range of HbA1c levels (5.7-6.4%) for diagnosing prediabetes (Figure 3).

The most frequently performed tests to screen for prediabetes in at-risk populations were fasting blood glucose (85.8%) and HbA1c (80.6%). In total, 25.1% used a two-hour oral glucose tolerance test, 24.5% utilized non-fasting blood glucose, and 1.9% did not routinely screen for diabetes. Only 33.5% of the participants were aware of the recommended minimum weight loss (5% of body weight) according to the ADA guidelines for patients with prediabetes. Overall, 56.1% had precise knowledge of the ADA-recommended minimum physical activity (150 minutes per week) for patients with prediabetes. The majority of participants (85.2%) used the ADA guidelines for diabetes screening (Table 2).

**Table 2:** Distribution of studied participants according to their response to test(s) ordered to screen for diabetes in at-risk populations, minimum weight loss, physical activity for prediabetics, and use of guidelines for diabetes screening. ADA: American Diabetes Association

Variable	Number (%)
Test(s) you order to screen for diabetes in at-risk populations in your practice (Select ALL that apply)	
Non-fasting blood glucose	38 (24.5)
Fasting blood glucose	133 (85.8)
Hemoglobin A1c	125 (80.6)
Two-hour oral glucose tolerance test	39 (25.1)
I do not routinely screen for diabetes	3 (1.9
Values that correspond to the lower limits of the laboratory criteria for diagnosing diabetes	
Mentioned the right range (100–125 mg/dL)	
True	86 (55.5)
False	69 (44.5)
Values that correspond to the lower limit of hemoglobin A1c (%) for diagnosing prediabetes	
Correct answer (5.7%)	79 (51)
Wrong answer	74 (47.7)
Don’t know	2 (1.3)
Values that correspond to the upper limit of hemoglobin A1c (%) for diagnosing prediabetes	
Correct answer (6.4%)	94 (60.6)
Wrong answer	60 (38.7)
Don’t know	1 (0.6)
Mentioned the right range (5.7–6.4%)	
True	67 (43.2)
False	88 (56.8)
Values that correspond to ADA recommendations for minimum weight loss (% of body weight) for patients with prediabetes	
Correct answer (5%)	52 (33.5)
Wrong answer	76 (49)
Don’t know	27 (17.4)
Values that correspond to ADA recommendations for minimum physical activity (minutes per week) for patients with prediabetes	
Correct answer (150)	87 (56.1)
Wrong answer	66 (42.6)
Don’t know	2 (1.3)
Guidelines, if any, you use for diabetes screening	
ADA	1 (0.6)
American Association of Clinical Endocrinologists	1 (0.6)
American Diabetes Association	132 (85.2)
US Task Force for Preventive Services	18 (11.6)
None	3 (1.9)

Of the study participants, 69.7%, 45.8%, and 52.9% strongly agreed that identifying prediabetes in their patients was important to manage health, to determine whether they needed to treat comorbid conditions, and to establish if elevated blood sugar levels required management, respectively (Table 3). In total, 40% strongly agreed that patients with prediabetes progress to diabetes more quickly than those with normoglycemia. Moreover, 65.8% and 43.9% strongly agreed that lifestyle modification or metformin, respectively, could reduce the risk of diabetes in patients with prediabetes.

**Table 3:** Participants’ opinions regarding the importance of identifying prediabetes in their patients, the condition representing a risk for the development of diabetes, and the use of lifestyle modifications and metformin to reduce the risk of diabetes.

	Strongly disagree	Disagree	Neutral	Agree	Strongly agree
Identifying prediabetes in my patients is:					
Important for managing their health	4 (2.6)	5 (3.2)	10 (6.5)	28 (18.1)	108 (69.7)
Helps me determine if I need to treat comorbid conditions, such as hypertension, more aggressively	5 (3.2)	12 (7.7)	25 (16.1)	42 (27.1)	71 (45.8)
Helps me determine if I need to treat elevated blood sugar levels	6 (3.9)	6 (3.9)	21 (13.5)	40 (25.8)	82 (52.9)
Patients with prediabetes progress to diabetes more quickly than those with normoglycemia	6 (3.9)	4 (2.6)	25 (16.1)	58 (37.4)	62 (40)
Lifestyle modification can reduce the risk of diabetes in my patients with prediabetes	5 (3.2)	5 (3.2)	11 (7.1)	32 (20.6)	102 (65.8)
Metformin can reduce the risk of diabetes in my patients with prediabetes	6 (3.9)	8 (5.2)	25 (16.1)	48 (31)	68 (43.9)

Regarding the initial management approach to prediabetes, 89% of the participants were able to identify that counseling patients on diet changes and physical activity to lose weight is the recommended initial management (Table 4). Additionally, 98.1% repeated the laboratory tests within a year in an individual recognized as having prediabetes; 49.7% saw the patient for follow-up after three months.

**Table 4:** Distribution of participants’ knowledge, practice, and beliefs regarding prediabetes management.

Variable	Number (%)
The best (recommended) initial management approach to a patient with prediabetes	
Discuss starting the patient on metformin	6 (3.9)
Provide counseling on diet changes and physical activity to lose weight (right answer)	138 (89)
Refer the patient for bariatric surgery	1 (0.6)
Refer the patient to a behavioral weight loss program	10 (6.5)
Your initial management approach to a patient with prediabetes in your practice and with your current resources (Select ALL that apply)	
I do not consider prediabetes a condition that requires specific management	8 (5.1)
Provide counseling on diet changes and physical activity to lose weight	142 (91.6)
Refer the patient to a nutritionist	87 (56.1)
Refer the patient to a behavioral weight loss program	69 (44.5)
Discuss starting the patient on metformin	81 (52.2)
Refer the patient for bariatric surgery	16 (10.3)
In a patient of yours that you diagnose with prediabetes, when, if at all, you have him/her repeat lab work	
Three months	68 (43.9)
Six months	33 (21.3)
No specific recommendation	4 (2.6)
One year (right answer)	47 (30.3)
Two years	3 (1.9)
In a patient of yours with prediabetes, when, if at all, you have him/her return for follow-up in your clinic	
Three months	77 (49.7)
Six months	36 (23.2)
No specific recommendation	5(3.2)
One year	31 (20)
Two years	6 (3.9)

There was an overall consensus that the barriers to lifestyle modification for individuals with prediabetes included patients’ lack of motivation (80%), physical limitations in doing an activity (71%), and lack of weight loss resources (61.9%). The most agreed interventions to improve management and treatment of prediabetes were improved access to diabetes prevention programs (82.5%) and more educational (80%) and improved nutrition (78.1%) resources for patients (Table 5).

**Table 5:** Participants’ opinions about barriers to lifestyle modification for their patients with prediabetes and interventions to improve management and treatment of prediabetes.

Variable	Strongly disagree	Disagree	Neutral	Agree	Strongly agree
Barriers to lifestyle modification for their patients with prediabetes					
Patients’ lack of motivation	5 (3.2)	2 (1.3)	24 (15.5)	69 (44.5)	55 (35.5)
Patients’ physical limitations in doing activity	5 (3.2)	10 (6.5)	30 (19.4)	68 (43.9)	42 (27.1)
Lack of weight loss resources for patients	8 (5.2)	19 (12.3)	32 (20.6)	64 (41.3)	32 (20.6)
Lack of nutrition resources for patients	7 (4.5)	18 (11.6)	37 (23.9)	56 (36.1)	37 (23.9)
Patients do not think it is important to make these changes	6 (3.9)	8 (5.2)	4 (26.5)	48 (31)	52 (33.5)
Financial limitations	21 (13.5)	19 (12.3)	45 (29)	49 (31.6)	21 (13.5)
Interventions to improve management and treatment of prediabetes					
More time for doctors to counsel patients	5 (3.2)	2 (1.3)	42 (27.1)	61 (39.4)	45 (29)
More educational resources for patients	1 (0.6)	5 (3.2)	25 (16.1)	58 (37.4)	66 (42.6)
Improved access to diabetes prevention programs (an evidence-based lifestyle change program)	3 (1.9)	3 (1.9)	21 (13.5)	63 (40.6)	65 (41.9)
Improved nutrition resources for patients	5 (3.2)	2 (1.3)	27 (17.4)	62 (40)	59 (38.1)
Improved access to weight loss programs	2 (1.3)	6 (3.9)	23 (14.8)	59 (38.1)	65 (41.9)
Improved access to bariatric surgery	7 (4.5)	13 (8.4)	47 (30.3)	49 (31.6)	39 (25.2)

Metformin was prescribed in individuals with the following parameters: body mass index ≥35 kg/m^2^ ( 72.2%), lack of response to lifestyle intervention (56.7%), history of gestational diabetes (44.5%), and HbA1c >6% (42.5%). However, 13.5% inferred that they did not believe in a rule for the use of metformin in prediabetes. Overall, 63.9% and 11% stated that they prescribed metformin to >5% and >50% of their prediabetes patients, respectively. Further, 68.4% of the participants indicated that the ADA guidelines were useful for the management of patients with prediabetes (Table 6).

**Table 6:** Participants responses’ to prescribing metformin for a patient with prediabetes and whether the American Diabetes Association guidelines for patients with prediabetes are helpful in managing patients with prediabetes.

Variable	Number (%)
Which of the following would make you more likely to prescribe metformin for a patient with prediabetes? (Select ALL that apply)	
I don’t believe in prescribing metformin for patients with prediabetes	21 (13.5)
BMI ≥35 kg/m^2^	110 (72.2)
Family history of diabetes	54 (34.8)
Dyslipidemia	49 (31.6)
Hypertension	44 (28.3)
History of gestational diabetes	69 (44.5)
HbA1c >6%	66 (42.5)
History of heart disease	42 (27)
Age <60	39 (25.1)
Age ≥60	38 (24.5)
Lack of response to lifestyle intervention	88 (56.7
Of your patients with prediabetes (without progression to diabetes), for what percentage have you prescribed metformin? (Select ONE)	
>25–50%	34 (21.9)
>5–25%	48 (31)
>50–75%	10 (6.5)
>75%	7 (4.5)
0	22 (14.2)
1–5%	34 (21.9
Have the American Diabetes Association guidelines for patients with prediabetes been helpful in managing your patients with prediabetes? (Select ONE)	
No, I’m familiar with them but they are not useful in my practice	7 (4.5)
No, I’m not familiar with them	14 (9)
Unsure	28 (18.1)
Yes	106 (68.4)

Barriers identified by participants toward the adoption of the ADA guidelines suggesting using metformin in certain patients with prediabetes included patients preferring not to take medication (63.9%), poor patient adherence (62.6%), providers’ lack of awareness of clinical guidelines for metformin (50.3%), and potential side effects (49.1%) (Table 7).

**Table 7:** Participants’ opinions about barriers to the adoption of the ADA guidelines which recommend using metformin in certain patients with prediabetes.

Variable	Strongly disagree	Disagree	Neutral	Agree	Strongly agree
Patients do not like taking medication	4 (2.6)	6 (3.9)	46 (29.7)	61 (39.4)	38 (24.5)
Medication cost to patient	19 (12.3)	44 (28.4)	52 (33.5)	32 (20.6)	8 (5.2)
Poor patient adherence	4 (2.6)	8 (5.2)	46 (29.7)	66 (42.6)	31 (20)
Potential side effects	4 (2.6)	17 (11)	58 (37.4)	57 (36.8)	19 (12.3)
Providers’ lack of awareness of clinical guidelines for metformin use	5 (3.2)	18 (11.6)	54 (34.8)	63 (40.6)	15 (9.7)
Lack of Food and Drug Administration approval for metformin use in prediabetes	18 (11.6)	42 (27.1)	58 (37.4)	31 (20)	6 (3.9)

## Discussion

This study was designed to evaluate prediabetes screening and treatment practices among PCPs and is the first national survey to address this subject in Saudi Arabia. This work has facilitated the recognition of gaps in PCPs’ knowledge regarding prediabetes which need to be addressed. Because patients with prediabetes have an increased risk of cardiovascular disease and all-cause mortality, early management of prediabetes, which includes the screening of eligible patients and interventions to prevent its progression to T2DM, is crucial [1,2].

There was a gap in participants’ knowledge of the risk factors for prediabetes; only 27.7% of the participants were aware of the nine risk factors included in the survey. There was also a gap in the recognition of the diagnostic cut-off for prediabetes because only 55.5% and 43.6% of the participants were aware of the fasting glucose (100-125 mg/dL) and HbA1c (5.7-6.4%) ranges, respectively. Some PCPs (24.5%) utilized non-fasting blood glucose to screen for diabetes, which is neither a parameter recommended by the ADA nor a component of the diagnostic criteria for prediabetes. This can lead to underscreening of eligible patients and underdiagnosis of prediabetes. Extensive national data regarding prediabetes is lacking in Saudi Arabia. However, in other countries, for example, the United States, the majority of individuals with prediabetes are not aware of their condition, a scenario potentially attributed to PCPs’ lack of knowledge of the diagnostic criteria [6].

This study also demonstrated a knowledge gap regarding the implementation of preventive measures. For instance, only 33.5% and 56.1% of PCPs were aware of the recommended ADA goals for minimum weight loss and physical activity, respectively, for patients with prediabetes. The knowledge of these parameters is important because these are evidence-based recommendations based on multiple studies that have demonstrated that lifestyle modifications successfully improved cardiometabolic markers and prevented progression from prediabetes to T2DM [1].

In other countries, PCPs reported barriers to prediabetes treatment related to the patients [8,13,14]. In this study, most of the PCPs described the following lifestyle modification barriers for their patients with prediabetes: physical limitations, poor motivation, lack of resources for nutrition and weight loss, and patients’ beliefs that lifestyle changes are not important. An approach that could be used to encourage individuals’ uptake of lifestyle modification is through counseling sessions with PCPs. This approach has been reported to be effective and engaging in changing patients’ perceptions toward lifestyle modifications [15-17].

Studies have shown that a very low proportion of patients with prediabetes take metformin [8,18]. Nevertheless, a previous survey of patients showed that the majority believe that lifestyle modification alone, metformin alone, or lifestyle modification combined with metformin are valid treatments for prediabetes [19]. Thus, many apparent barriers to the adoption of the ADA guidelines regarding the use of metformin reported by PCPs in some individuals with prediabetes in this study might be false. The data from this survey showed that in Saudi Arabia, PCPs prescribe metformin for less than half of their patients with prediabetes.

Suggested interventions that could improve the management and treatment of prediabetes reported by PCPs were similar to those published in studies from other countries, such as improved patient access to diabetes prevention programs, educational resources, and nutritional resources [6,7].

This study has multiple strengths. It is the first national survey in Saudi Arabia to evaluate PCPs’ knowledge and practices concerning prediabetes. Responses were received from PCPs from the majority of the principal territories within Saudi Arabia and a spectrum of practice types. Limitations of this study include the possibility of recall and social desirability bias. Another important limitation is that most of the study participants were family medicine specialists and resident physicians, which could limit data generalizability to other specialties and other training levels. Moreover, the majority of respondents were Saudi, limiting the generalizability of the findings to non-Saudi PCPs who constitute a large portion of PCPs in Saudi Arabia.

## Conclusions

This national survey uncovered important gaps in PCPs’ knowledge regarding prediabetes in Saudi Arabia, which can contribute to both underscreening and undertreating prediabetes. The identification of these gaps is essential to focus educational endeavors toward PCPs who act as first-line healthcare providers and screen for and manage prediabetes and diabetes. Addressing their knowledge gaps can have a substitutional effect on the fight against diabetes.

## Appendices

The survey used in this study

Part I. The following questions apply to your knowledge and practices regarding diabetes screening.

1.     Which of the following are risk factors that might prompt you to screen for diabetes? (Select ALL that apply)

      Age ≥ 45

      BMI ≥25 kg/m2

      Hypertension

      Dyslipidemia

      Heart disease

      Family history of diabetes in a first-degree relative

      Sedentary lifestyle

      History of gestational diabetes

      Smoking

2.     In your practice, which test(s) do you order to screen for diabetes in at-risk populations? (Select ALL that apply)

      Non-fasting blood glucose

      Fasting blood glucose

      Hemoglobin A1c

      Two-hour oral glucose tolerance test

      I do not routinely screen for diabetes

3.     Please circle the values that correspond to the lower limits of the lab criteria for diagnosing diabetes.

a.     Fasting glucose (mg/dL):                                   Don’t know

100  102  104  106  108  110  112  114  116  118  120  122  124  126  128  130  132  134  136  138  140

b.     Hemoglobin A1c (%):                                        Don’t know

5.0    5.1    5.2    5.3    5.4    5.5    5.6    5.7    5.8    5.9   6.0   6.1   6.2   6.3   6.4   6.5   6.6   6.7   6.8   6.9   7.0

4.     Please circle the values that correspond to the upper and lower limits (range) of the laboratory criteria for diagnosing prediabetes.

a.     Fasting glucose (mg/dL):                                   Don’t know

70    75    80    85    90    95    100    105    110    115    120    125    130    135    140    145    150    155    160

b.     Hemoglobin A1c (%):                                        Don’t know

5.0    5.1    5.2    5.3    5.4    5.5    5.6    5.7    5.8    5.9   6.0   6.1   6.2   6.3   6.4   6.5   6.6   6.7   6.8   6.9   7.0  

5.     Please circle the value that corresponds to ADA recommendations for lifestyle modification for patients with prediabetes:

a.     Minimum weight loss (% of body weight):                     Don’t know

1      2      3       4       5       6      7      8      9      10      11      12      13      14      15      16      17      18      19      20

b.     Minimum physical activity (minutes per week):             Don’t know

10    20    30    40    50    60    70    80    90    100    110    120    130    140    150    160   170   180   190   200

6.     Which guidelines, if any, do you use for diabetes screening? (Select ONE)

      None

      American Diabetes Association

      US Task Force for Preventive Services

      American Association of Clinical Endocrinologists

      Other ____________________________

**Table 8:** Questions 7-10 of the survey.

How strongly do you agree or disagree with the following statements?					
	Strongly disagree	Disagree	Neutral	Agree	Strongly agree
7. Identifying prediabetes in my patients is:					
a. Important for managing their health	□	□	□	□	□
b. Helps me determine if I need to treat comorbid conditions such as hypertension more aggressively	□	□	□	□	□
c. Helps me determine if I need to treat elevated blood sugars	□	□	□	□	□
8. Patients with prediabetes progress to diabetes more quickly than those with normoglycemia	□	□	□	□	□
9. Lifestyle modification can reduce the risk of diabetes in my patients with prediabetes	□	□	□	□	□
10. Metformin can reduce the risk of diabetes in my patients with prediabetes	□	□	□	□	□

Part II. The following questions apply to your knowledge, practices, and beliefs regarding prediabetes management.

1.     Which of the following is the best (recommended) initial management approach to a patient with prediabetes? (Select ONE)

      Provide counseling on diet changes and physical activity to lose weight

      Refer the patient to a behavioral weight loss program

      Discuss starting the patient on metformin

      Refer the patient for bariatric surgery

      Other _________________________________

2.     In your practice and with your current resources, what is your initial management approach to a patient with prediabetes? (Select ALL that apply)

      I do not consider prediabetes a condition that requires specific management

      Provide counseling on diet changes and physical activity to lose weight

      Refer the patient to a nutritionist

      Refer the patient to a behavioral weight loss program

      Discuss starting the patient on metformin

      Refer the patient for bariatric surgery

      Other__________________________________

3.     In a patient of yours that you diagnose with prediabetes, when, if at all, would you have him/her repeat lab work? (Select ONE)

      3 months

      6 months

      One year

      Two years

      No specific recommendation

      Other_____________________________

4.     In a patient of yours with prediabetes, when, if at all, would you have him/her return for follow up in your clinic? (Select ONE)

      3 months

      6 months

      One year

      Two years

      No specific recommendation

      Other____________________________

**Table 9:** Questions 15 and 16 of the survey.

15. How strongly do you agree or disagree that the following are barriers to lifestyle modification for your patients with prediabetes?					
	Strongly disagree	Disagree	Neutral	Agree	Strongly agree
Patient’s lack of motivation	□	□	□	□	□
Patient’s physical limitations in doing an activity	□	□	□	□	□
Lack of weight loss resources for patient	□	□	□	□	□
Lack of nutrition resources for patient	□	□	□	□	□
Patients don’t think it is important to make these changes	□	□	□	□	□
Financial limitations	□	□	□	□	□

16. How strongly do you feel that these interventions will improve the management and treatment of prediabetes?					
	Strongly disagree	Disagree	Neutral	Agree	Strongly agree
More time for doctors to counsel patients	□	□	□	□	□
More educational resources for patients	□	□	□	□	□
Improved access to diabetes prevention programs (an evidence-based lifestyle change program)	□	□	□	□	□
Improved nutrition resources for patients	□	□	□	□	□
Improved access to weight loss programs	□	□	□	□	□
Improved access to bariatric surgery	□	□	□	□	□

Part III. The following questions apply to your knowledge, practices, and beliefs regarding the treatment of prediabetes with medication.

17.  Which of the following would make you more likely to prescribe metformin for a patient with prediabetes? (Select ALL that apply)

      I don’t believe in prescribing metformin for patients with prediabetes

      BMI ≥35 kg/m2

      Family history of diabetes

      Dyslipidemia

      Hypertension

      History of gestational diabetes

      HbA1c >6%

      History of heart disease

      Age <60

      Age ≥60

      Lack of response to lifestyle intervention

      Other _________________________________

18.  Of your patients with prediabetes (without progression to diabetes), for what percentage of them have you prescribed metformin? (Select ONE)

      0%

      1-5%

      >5-25%

      >25-50%

      >50-75%

      >75%

19.  Have the American Diabetes Association guidelines for patients with prediabetes been helpful in managing your patients with prediabetes? (Select ONE)

      Yes

      No, I’m not familiar with them

      No, I’m familiar with them but they are not useful in my practice

      Unsure

**Table 10:** Question 20 of the survey.

20. The ADA guidelines suggest using metformin in certain patients with prediabetes. Studies suggest that few patients are prescribed metformin for prediabetes. How strongly do you agree or disagree that the following are barriers to adoption?					
	Strongly disagree	Disagree	Neutral	Agree	Strongly agree
Patients do not like taking medications	□	□	□	□	□
Medication cost to patients	□	□	□	□	□
Poor patient adherence	□	□	□	□	□
Potential side effects	□	□	□	□	□
Providers’ lack of awareness of clinical guidelines for metformin use	□	□	□	□	□
Lack of FDA approval for metformin use in prediabetes	□	□	□	□	□
Other reason:					

Part IV. Demographic information

21.  What is your gender?

      Female

      Male

22.  What is your nationality?

      Saudi

      Non-Saudi, please specify _____________________

23.  What is your specialty? (Select ALL that apply)

      Family Medicine

      Internal Medicine

      OB/Gyn

      General practitioner (without advanced training)

      Other______________________________

24.  What is your current level of training?

      Consultant

      Specialist

      Fellow

      Resident

      General practitioner (without advanced training)

25.  Please select the type of organization you work with now (Select ALL that apply).

      University

      Ministry of health

      National guard

      Military

      Private

      Other______________________

26.  Please select the city of your current practice.

      Jeddah

      Riyadh

      Dammam

      Makkah

      Al Madinah

      Other ______________________________

27.  Please select country/countries where you did any level of your postgraduate training (Select ALL that apply.

      Saudi Arabia

      United States

      Canada

      United Kingdom

       Germany

      France

      Egypt

      Other_______________________________
